# An ultrasensitive sensing strategy based on CRISPR/Cas13a and T7 RNA polymerase amplification for detection of extracellular vesicles

**DOI:** 10.1007/s44211-025-00828-3

**Published:** 2025-07-17

**Authors:** Fengying Ran, Huimin Huang, Bing Shang, Weidong Peng, Lun Wu, Kang Ling, Xiaoyu Xie

**Affiliations:** 1 School of Pharmacy, Health Science Center, Xi’an Jiaotong University, Xi’an, 710061 Shanxi China; 2 Sinopharm Dongfeng General Hospital, Hubei University of Medicine, Shiyan, 442008 Hubei China; 3 Department of Pharmacy, National Cancer Center/National Clinical Research Center for Cancer/Cancer Hospital, Chinese Academy of Medical Sciences and Peking Union Medical College, Beijing, 100021 China

**Keywords:** Extracellular vesicles, T7 RNA polymerase, CRISPR/Cas13a, Aptamer, Lung cancer

## Abstract

Extracellular vesicles (EVs) are important biomarkers for an early diagnosis of lung cancer. Herein, we proposed an ultrasensitive fluorescent sensing platform for EVs detection, which involves aptamer and streptavidin-modified magnetic nanoparticles (SA-MB) magnetic separation technology as well as T7 RNA polymerase-assisted CRISPR/Cas13a system, which can achieve target recycling signal amplification. In this detection method, biotin-modified CD63 aptamer hybridizes first with the aptamer Blocker (T7 promoter) and then binds to SA-MB. When adding EVs, the CD63 aptamer in CD63 aptamer/Blocker/SA-MB complex captures EVs causing the release of Blocker single chain. Subsequently, large amounts of ssRNAs, which are generated with the assistance of Blocker-initiated T7 RNA polymerase, were recognized by CRISPR/Cas13a and trigger its trans-cleavage report probe (F-Q). Eventually, the report probe labeled with fluorescent dye (FAM) and quench group (BHQ) at both ends was cut to produce fluorescent signal. The designed sensor combined this with a signal amplification strategy based on T7 RNA polymerase and CRISPR/Cas13a to significantly enhance the sensitivity and specificity of EVs detection. The use of magnetic separation technology eliminates interference from complex matrices and improves EVs detection efficiency, while the introduction of T7 RNA polymerase and CRISPR/Cas13a enables multiple amplifications of the sensor signals, and enhancing the accuracy and sensitivity of the method. Ultimately, the combination of multiple amplification reactions resulted in a detection limit (LOD) for EVs as low as 60 particles/mL (approximately 1 zmol/L). In addition, this detection method can specifically distinguish EVs from other confounding substances and efficiently detect plasma EVs from lung cancer and healthy individuals in actual samples. Indicating this sensing platform is a valuable tool for early lung cancer detection.

**Graphical abstract:**

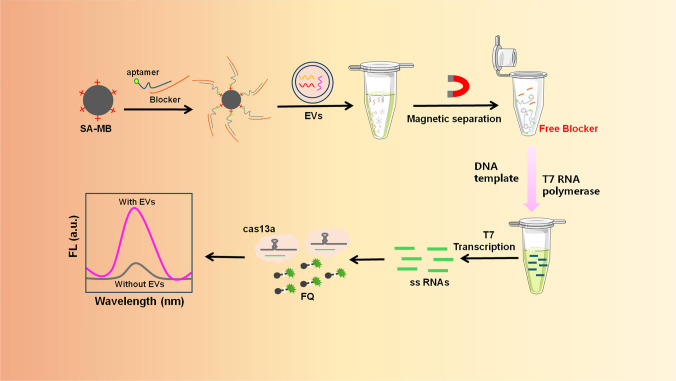

**Supplementary Information:**

The online version contains supplementary material available at 10.1007/s44211-025-00828-3.

## Introduction

Lung cancer is one of the most common causes of cancer-related death worldwide, which seriously threatens human health. According to global cancer statistics, lung cancer was the most frequently diagnosed cancer in 2022 (2480301, 12.4%) [1]. The incidence and mortality of lung cancer in China rank first among malignant tumors. Due to the lack of specific clinical symptoms in the early stages, most lung cancer patients are already in the late stage at the initial diagnosis, missing the best treatment opportunity and having a low 5-year survival rate, suggesting the importance of early diagnosis [2, 3]. However, conventional methods, such as computed tomography (CT) and histopathological biopsy, which have been widely used in the screening of lung cancer, lack the sensitivity and specificity of early detection [4, 5]. With the advantages of simple specimen collection, high repeatability and minimally invasive, liquid biopsy can be used for accurate treatment, prognosis evaluation, and curative effect monitoring of lung cancer [6]. So far, various biomolecules, such as ctDNA, miRNA, circulating tumor cells (CTCs), proteins, and extracellular vesicles (EVs), have been utilized as biomarkers for early diagnosis in tumor liquid biopsy [7, 8]. EVs are produced by living cells in body fluids, can encapsulate and transfer various parental cell biomarkers, and serve as important communication carriers between cells [9]. Compared with other liquid biomarkers, EVs exhibit great promise due to incomparable advantages including high abundance, great stability, rich content, and longitudinal sampling [10]. Therefore, it is urgent to establish an accurate, simple, and sensitive method with circulating EVs as biomarkers.

Up till now, various detection platforms have been applied to determine EVs, including electrochemical impedance spectroscopy (EIS) [11], electrochemical [12], colorimetry and photothermal [13], surface-enhanced Raman spectroscopy (SERS) [14], surface plasmon resonance (SPR) [15], nanopore [16], flow cytometry [17], and enzyme-linked immunosorbent assay (ELISA) [18]. However, many shortcomings, such as complex operation, expensive instruments, time-consuming, and limited sensitivity, limit its application [19]. Fluorescence-based biosensors stand out for their advantages of simple operation and low cost [20]. However, because of the complex matrix interference in blood and the ultra-low abundance of EVs in the early stage of disease, the method of accurately detecting EVs in blood based on fluorescence is still challenging. Consequently, establishing a straightforward and efficient approach to enhance both the sensitivity and specificity of the EVs fluorescence detection platform holds considerable importance.

The clustered regularly interspaced short palindromic repeats (CRISPR) systems, initially emerged as gene editing technology, have attracted wide attention due to its simplicity and high efficiency [21]. As a member of the CRISPR systems, CRISPR/Cas13a has RNA-directed RNA cleavage ability in which RNA-guided trans-endonuclease activity is highly specific and efficiently activated when the target RNA has a complementary sequence to the crRNA [22, 23]. The activated Cas13a enzyme possesses the ability to facilitate the degradation of a wide array of non-specific RNA molecules [24]. Thus, the CRISPR/Cas13a is expected to be a powerful tool for assisting biosensor construction. Over the past decade, a large number of detection platforms based on CRISPR/Cas13a had been established for detecting viruses (such as HBV, H7N9, CPV-2, SARS-CoV-2), food-borne pathogens [25–28], DNA, RNA, miRNA, and protein [29–31]. Nevertheless, CRISPR/Cas13a-based fluorescence detection sensor still has problems such as insufficient sensitivity. Therefore, in order to improve the sensitivity of the biosensors, it is necessary to introduce a signal amplification strategy.

T7 RNA polymerase is a DNA-dependent RNA polymerase, which is highly specific to the T7 promoter and catalyzes the synthesis of 5 '→ 3' RNA downstream of single-stranded DNA or double-stranded DNA from the promoter [32–34]. T7 promoter derived from T7 phage can react specifically with T7 RNA polymerase and starts the transcription of T7 phage gene [35]. By employing double-stranded DNA that incorporates the T7 promoter sequence as a template and utilizing nucleoside triphosphates (NTPs) as substrates, T7 RNA polymerase is capable of generating RNA that is complementary to the downstream single-stranded DNA [36, 37]. The capacity of powerful molecular amplification has led to T7 RNA polymerase being widely used in the construction of various biosensors [38]. Consequently, the integration of T7 RNA polymerase with a CRISPR/Cas13a-mediated signal amplification approach could significantly enhance the amplification efficiency of EVs.

Herein, we present a fluorescent-sensing platform based on CRISPR/Cas13a–streptavidin magnetic beads aptamer and T7 RNA polymerase-assisted amplification for the detection of EVs derived from lung cancer. In this method, the streptavidin- modified magnetic nanoparticles (SA-MB) have good manipulation performance under the action of external magnetic field and can be used to separate and purify samples to reduce matrix interference [39]. In addition, CRISPR/Cas13a has a target-specific induced accessory enzyme activity, which is widely used in the field of biosensor [40]. Aptamer is a nucleic acid sequence that can specifically bind to the target (cells, EVs, proteins and small molecules) [41, 42]. The aptamer of EVs surface protein CD63 is first hybridized with its complementary sequence and then modified on SA-MB. In the presence of EVs, the CD63 aptamer binds to the CD63 protein on the surface of EVs and releases its complementary chain. Following this, the liberated complementary single strand, which encompasses the T7 promoter sequence, associates with the DNA template strand. In the presence of T7 RNA polymerase, it facilitates the replication of numerous RNA strands. Concurrently, the generated RNA strand is recognized by the CRISPR/Cas13a system, which activates its trans-cleavage report probe (F–Q). As a result, the report probe, which is tagged with a fluorescent dye (FAM) and a quenching group (BHQ) at either terminus, undergoes cleavage, thus producing a notable fluorescence signal that is directly proportional to the concentration of EVs captured by SA-MB. They developed a method that selectively captures EVs using CD63 aptamers and Blocker, and use of magnetic separation technology eliminates interference from complex matrices and improves EVs detection efficiency, and combined this with a signal amplification strategy based on T7 RNA polymerase and CRISPR/Cas13a to significantly enhance the accuracy and sensitivity of the biosensor. Ultimately, this method can accurately distinguish lung cancer patients from healthy individuals using clinical samples, suggesting its potential for cancer diagnosis. Moreover, the method can be extended to the detection of other biomarkers by selecting appropriate aptamers.

## Experimental section

### Reagents and materials

LwaCas13a was purchased from New England Biolabs Ltd. (New England, USA), SA-MB (Dynabeads™ M-280 Streptavidin, diameter of 2.8 μm), T7 RNA polymerase, and NTPs were purchased from Thermo Fisher Scientific Co., Ltd. in Waltham, MA, USA., and the oligonucleotides used in this work were synthesized by the Shanghai Sangon Biotech Co., Ltd (Shanghai, China), with the sequences listed in the supplementary information (Table S1). The filters (0.22 μm) were acquired from Millipore Corp. (Bedford, MA, USA), and the proteins PD-L1, EpCAM, cTnI, and CEA were purchased from Abcam Biotechnology Co. Ltd (Shanghai, China). Human sera were provided by Affiliated Dongfeng Hospital, Hubei University of Medicine, and approved by Hospital’s Ethics Committee. TE buffer, PBS, TBE, DEPC-treated water, ddH_2_O, Tris–HCl, EDTA, NaCl, and other regents of analytical grades were obtained from Servicebio Biotechnology (Wuhan, China).

### Cell culture and EVs isolation

A549 cells were inoculated in the 75 mL culture flask and maintained in Dulbecco's modified Eagle's medium (DMEM), supplemented with 10% fetal bovine serum (FBS), under a humidified environment at 37 °C with 5% CO_2_. Following this, the cell supernatant was harvested after a 48-hour incubation period in a serum-free medium (approximately 4 × 10^7^ cells). Using the current considered gold standard ultracentrifugation method, EVs were extracted from a tumor cell supernatant culture medium. Specific extraction steps are as follows: (1) centrifugation at 300 g for 10 min to remove floating cell sediments; (2) centrifugation at 2000 g, 4 °C of the supernatant for 20 min, and then pass it through a 0.22 μm filter membrane; (3) centrifugation at 10,000 g of filtered filtrates for 30 min at 4 °C, subsequently, in order to improve the separation purity of exosomes, and the supernatant was ultra-centrifuged and discarded after 1,10,000 g at 4 °C for 70 min; (4) add 1 mL PBS to re-suspend and make up to 25 mL. After ultracentrifugation at 1,10,000 g for 70 min again at 4 °C, 200 μL PBS was re-suspended and precipitated, and stored at – 80 ℃ for future use. In order to further verify whether the EVs were successfully isolated, the obtained samples were characterized by transmission electron microscopy (TEM), nanoparticle tracking analysis (NTA), and Western blotting (WB). The detailed procedure is given in Supporting Information.

### EVs determination procedure

In our design, the EVs were first captured by CD63 aptamers and released Blocker single chains through chain displacement reactions. In the subsequent steps, the released inhibitor single-stranded promotes the synthesis of a large amount of single-stranded RNA (ssRNA) in the presence of T7 RNA polymerase and DNA template chains. Ultimately, these synthesized ssRNAs can be recognized by the CRISPR/Cas13a system, which activates its trans-cleavage reporter probe (F-Q) for subsequent fluorescence detection.

#### EVs capture

First, we performed the capture EVs using CD63 aptamers/T7 Blocker double strand. 5 μL CD63 aptamer, 7.5 μL T7 Blocker, and 37.5 μL PBS buffer were mixed. Then, pre-hybridized with a PCR machine to form Apt63 (95℃ for 5 min, cooled down to temperature). After that, wash SA-MB (10 μL, 10 mg/mL) three washing cycles using a washing buffer. Following this, 100 μL of binding buffer was incorporated into the mixture. Apt63 (10 μL) was added to the diluted SA-MB solution, which was then vigorously mixed at 37 ℃ for 30 min. After this incubation period, magnetic separation was employed to isolate the Apt63-labeled SA-MBs, which were further washed three times with a washing buffer. Add 100 μL of EVs with different concentrations into Apt63-labeled SA-MBs and shake vigorously at 37 ℃ for 1 h. After magnetic separation, a supernatant containing T7 Blocker was obtained.

#### T7 RNA polymerase amplification reaction

Subsequently, 2 μL of a T7 blocking chain product was introduced into 18 μL of the amplification reaction solution designed for T7 RNA polymerase-mediated amplification. This reaction mixture comprised the following components: 2 μL of 10× transcription buffer, 1 μL of NTP Mix (10 mM), 2 μL of DNA template (2 μM), 2.5 μL of T7 RNA Polymerase (50 U), 1 μL of RNase inhibitor (20 U), and 9.5 μL of RNase-free water. Then incubate the solution at 37 ℃ for 3 h to promote transcription amplification process, and this is the amplification technology of T7 RNA polymerase, which can generate a large amount of single-stranded RNA (ssRNA) products, thereby achieving signal amplification.

#### CRISPR/Cas13a-based fluorescence signal measurement

First, Cas13a was mixed well with crRNA and placed them on ice for 10 min to form a Cas13a/crRNA complex. Next, 20 μL of CRISPR/Cas13a reaction system was added into the tube, which included 2 μL expand product (ssRNA), 2 μL 10× Cas13a reaction buffer, 100 nM Cas13a/crRNA, and 10 μM F-Q probe. All ingredients were mixed evenly and performed Cas13a-mediated F-Q probe trans-cleavage reaction at 37℃ for 40 min. Subsequently, 480 μL of water treated with diethyl pyrocarbonate (DEPC) was incorporated into the reaction mixture. The fluorescence intensity of the resultant solution was subsequently quantified employing a fluorescence spectrophotometer (Hitachi, F-4700, Japan. Excitation/emission: 5 nm). Finally, the quantitative expression of EVs was shown by fluorescence intensity.

### Detection in clinical EVs samples

Initially, blood samples of 2 mL each were collected from a cohort comprising 10 patients diagnosed with lung cancer and 10 healthy individuals. Subsequently, these samples were subjected to centrifugation at 3000 g for 5 min. Following centrifugation, the supernatant was carefully aspirated and subsequently filtered using micropores with a diameter of 0.22 μm. Plasma EVs were extracted following previous ultracentrifugation steps. Finally, 200 μL PBS was added to dilute the pellet to obtain EVs, and stored at -80 ℃ for standby. Subsequently, the sensing method designed in this study was used to detect EVs.

## Results and discussion

### Principle of the proposed method

**Fig. 1:**
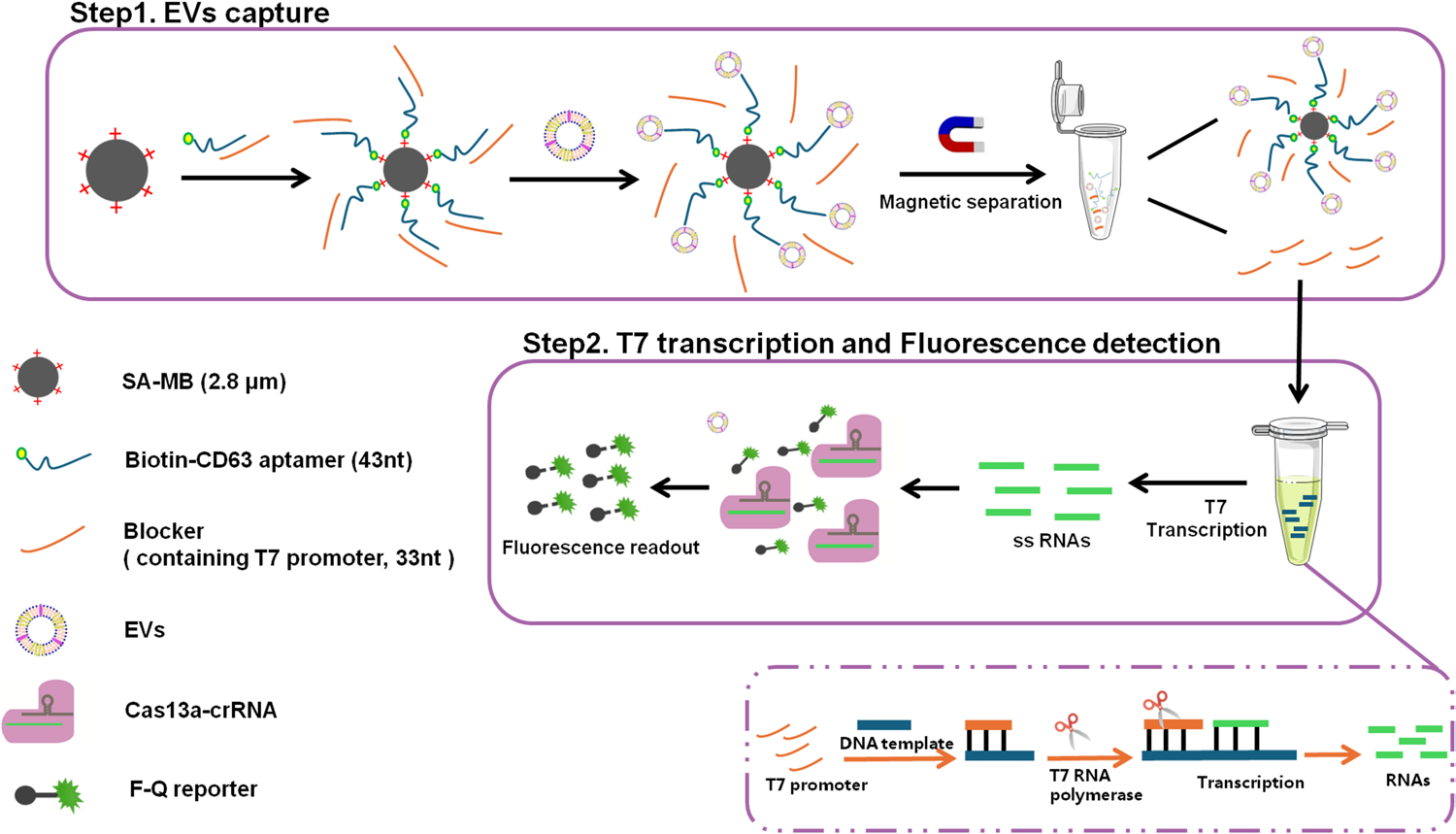
Fluorescence detection of EVs using CRISPR/Cas13a

Principle of the proposed sensor is illustrated in Fig. 1, including EVs capture, T7 transcription, and Cas13a detection procedure. First, due to the CD63 protein expressed on the surface of the EVs [43], the biotin-modified CD63 aptamer is complementary with the aptamer Blocker (T7 promoter) to form the duplex of CD63 aptamer/Blocker, and then binds to the SA-MB. After adding EVs, the CD63 aptamer in CD63 aptamer/Blocker/SA-MB complex captures EVs, causing the release of a single-chain Blocker. After magnetic separation, the solution containing the released Blocker is mixed with the T7 RNA polymerase reaction system. Subsequently, a large amount of RNA was transcribed and amplified in vitro, further activating the activity of Cas13a. As a result, the reporter probe (F–Q) labeled with fluorescent dye (FAM) and quenching group (BHQ) at both ends was cleaved, generating a fluorescent signal. Because each activated CRISPR/Cas13a molecule can cleave a large number of reporter genes within 20 minutes, the originally detected signal can be amplified rapidly to achive rapid and sensitive detection of EVs.

### Characterization of isolation EVs

**Fig. 2:**
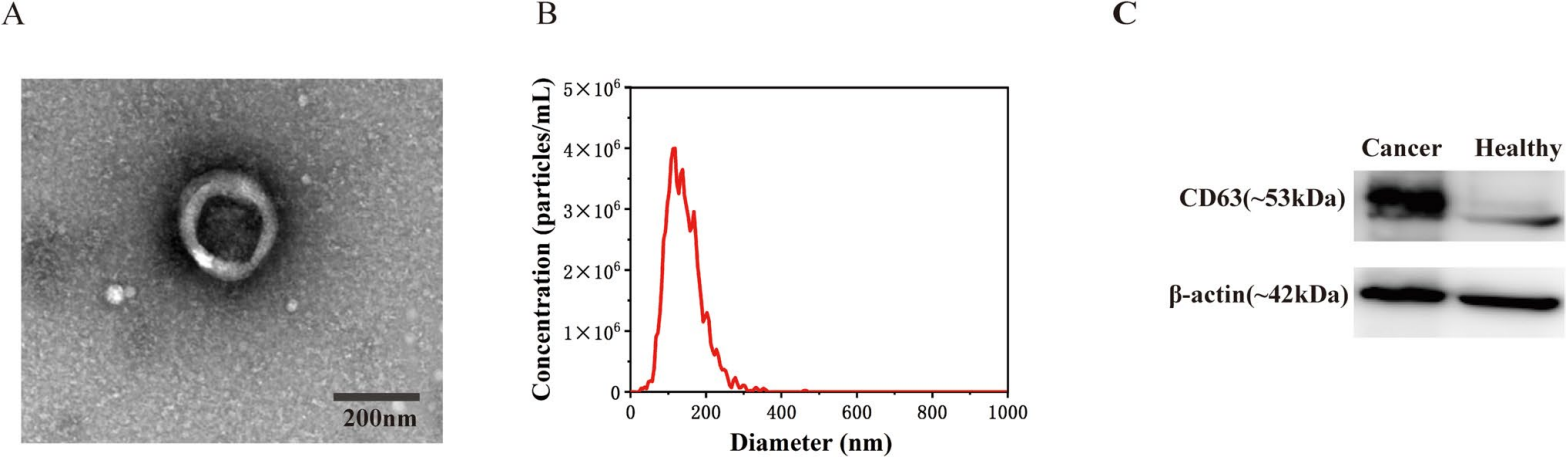
Characterization of EVs; **A** TEM image, **B** NTA analysis, **C** western blotting analysis of the cancer and healthy EVs marker CD63 and β-actin

To confirm the successful separation of EVs from a cell culture medium, the characteristic morphology, particle size distribution, and proteins of EVs were, respectively, measured by TEM, NTA, and western blotting. As illustrated in Fig. 2A, the EVs exhibited a characteristic morphology characterized by a cup-shaped double membrane structure, with an average diameter of around 100 nanometers, confirming successful separation of EVs [44]. Subsequently, NTA data revealed that the EVs concentration was 1.6 × 10^10^ particles/mL, with the particle size distribution ranges from 40 to 200 nm and are centered at 114 nm (Fig. 2B). The surface marker CD63 protein was significantly detected in EVs derived from cancer cells and healthy groups, and the expression of CD63 was higher in cancer cells (Fig. 2C).

### Feasibility analysis of methods

**Fig. 3:**
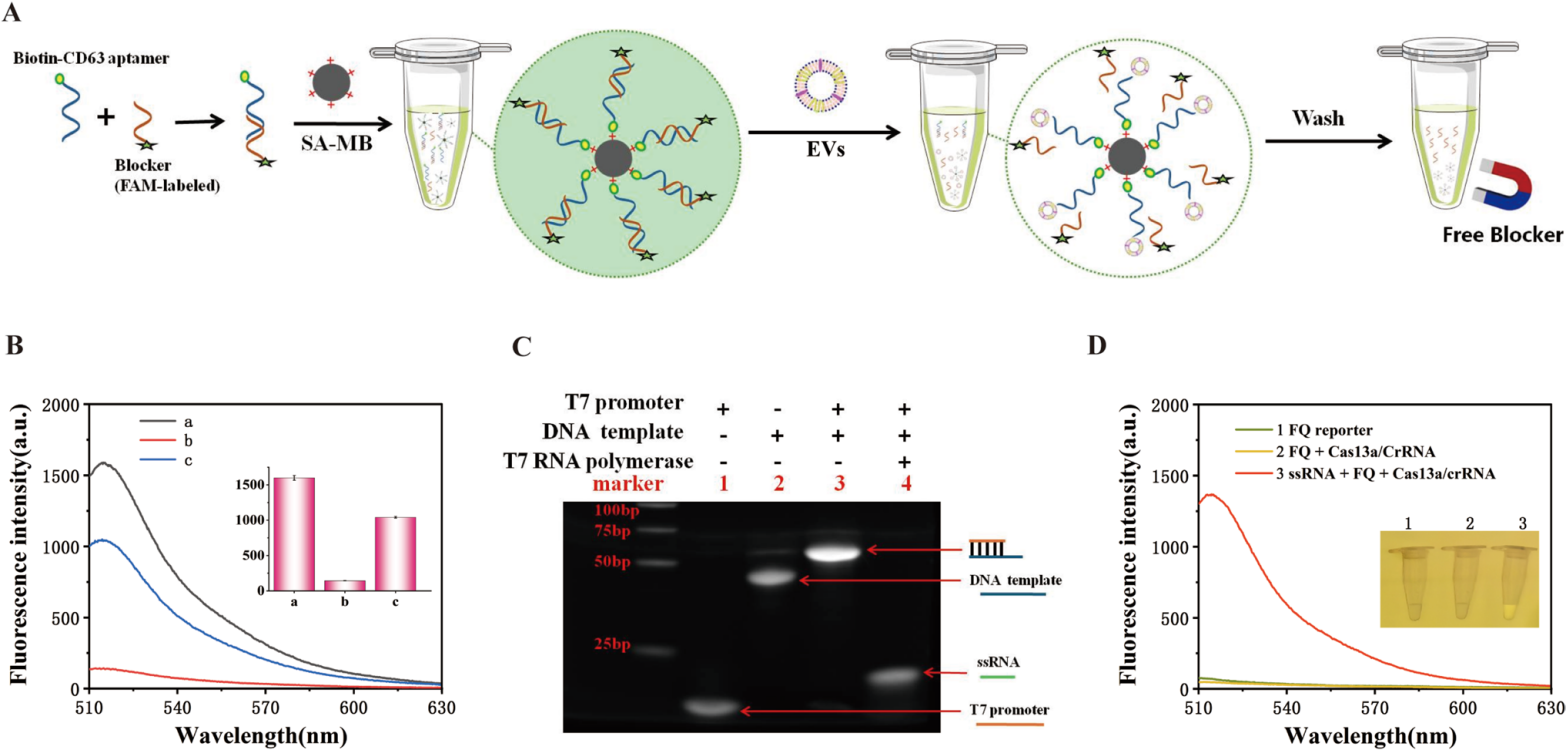
The sensor of feasibility. **A** Schematic illustration of EVs replacing Blocker process based on SA-MB. **B** Fluorescence spectra under different reaction conditions. line a: CD63 aptamer + Blocker (FAM-labeled); line b: CD63 aptamer + Blocker (FAM-labeled) + SA-MB (supernatant after SA-MB); line c: CD63 aptamer + Blocker (FAM-labeled) + SA-MB + EVs (supernatant after SA-MB). CD63 aptamer (55nt): 5 μM, Blocker (33nt): 7.5 μM, SA-MB (diameter of 2.8 μm): 1 mg/mL, EVs: 3.5*10^4^ particles/mL. **C** Polyacrylamide gel electrophoresis analysis of T7 promoter-induced transcription amplification. **D** Fluorescence spectra induced by different samples reactions: line 1: FQ-reporter; line 2: FQ-reporter + Cas13a/crRNA; line 3: FQ-reporter + Cas13a/crRNA + ssRNA(T7 transcription products)

Feasibility of the sensor was investigated as shown in Fig. 3. First, as shown in Fig. 3A, the feasibility of EVs replacing Blocker based on SA-MB was investigated. As shown in Fig. 3B, curve a, high fluorescence signal (about 1500 a.u) of FAM-labeled Blocker was observed, then the mixture of CD63 aptamer, FAM-labeled Blocker, and SA-MB was placed on an oscillator and mixed for 40 min. Subsequent to the magnetic separation process, the fluorescence signal present in the supernatant can be disregarded (refer to Fig. 3B, curve b), indicating complete binding of FAM-labeled Blocker/CD63 aptamer double chain to SA-MB. Interestingly, the fluorescence signal of the supernatant was restored when the FAM-labeled Blocker/CD63 aptamer/SA-MB complex was mixed with the EVs for 1 h (Fig. 3B, curve c). These results indicate that successful recognition and binding of exosomes and CD63 aptamer in the FAM-labeled Blocker/CD63 aptamer/SA-MB complex released the free FAM-labeled Blocker.

As shown in Fig. 3C, the transcriptional amplification strategy induced by T7 promoter was also verified by 15% non-denaturing PAGE. The T7 promoter and the corresponding DNA template exhibited distinct single bands in lanes 1 and 2. In contrast to the T7 promoter, DNA template showed lower electrophoretic mobility due to the long DNA template chain (55nt). In the absence of T7 RNA polymerase, T7 promoter/template duplex showed lower electrophoretic mobility (lane 3). In contrast, when T7 RNA polymerase was present, a higher electrophoretic migration rate was noted in comparison to the double-stranded DNA template/T7 promoter (lane 4), indicating that T7 promoter successfully induced transcriptional amplification to produce a large number of ssRNAs.

Finally, to verify the recognition of the amplified ssRNA by crRNA-guided CRISPR/Cas13a system, transcripts and F-Q reporter genes were incubated with Cas13a/crRNA for 40 minutes to detect the fluorescence intensity. As shown in Fig. 3D, significant fluorescence enhancement was observed only in the presence of ssRNA. This indicates that the hybridization of transcriptional ssRNA with crRNA activated cas13a trans-endonuclease activity, and therefore F-Q reporter gene degradation.

### Optimization of reaction conditions

**Fig. 4:**
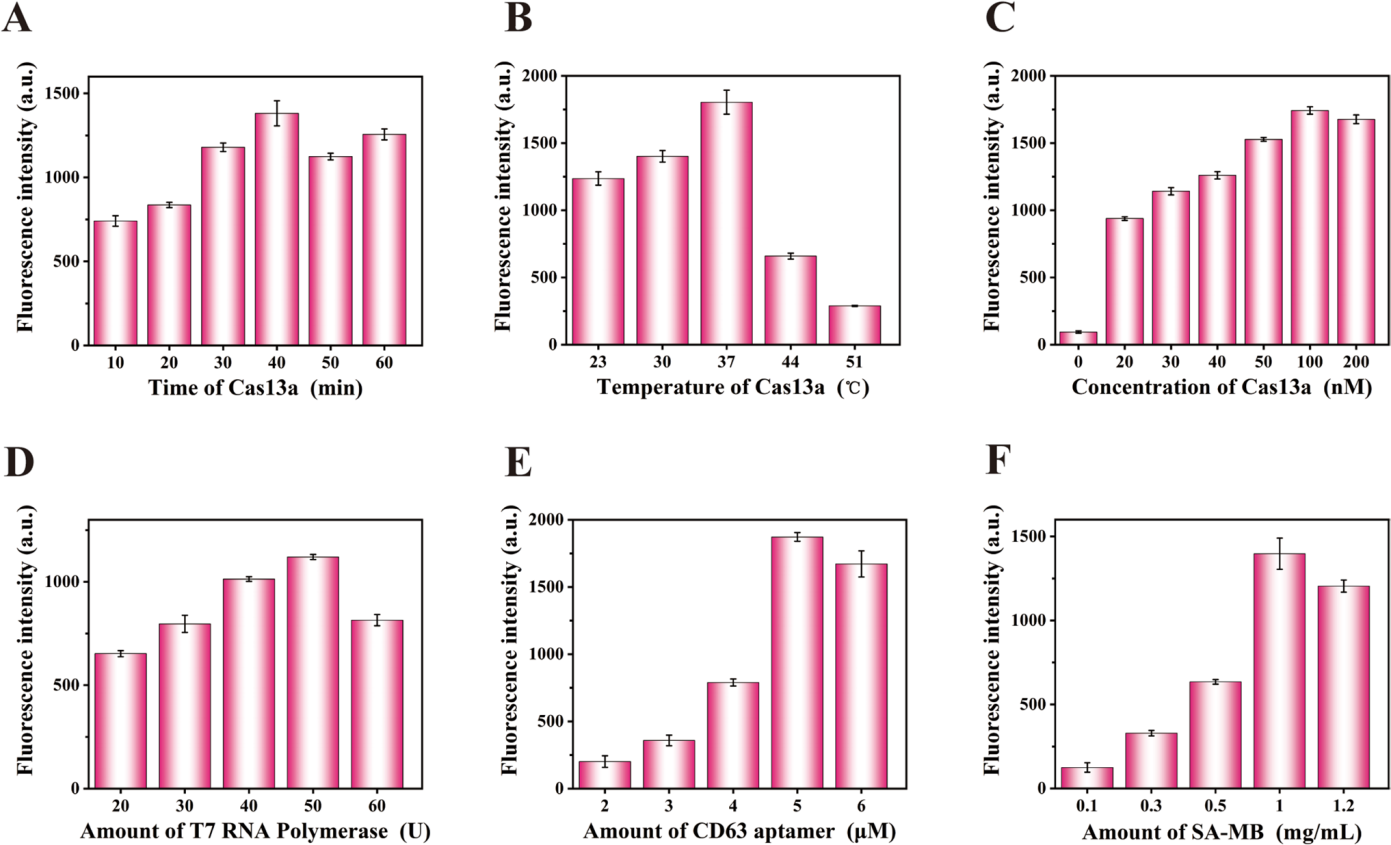
Optimization of various reaction conditions. **A**–**C** Under different time, temperature and concentration of cas13a. **D** Under different amount of T7 RNA polymerase. **E** Under different amount of CD63 aptamer. **F** Under different amount of SA-MB. a.u. arbitrary units, the error bar represents the standard deviations of measurements (*n* = 3). The concentration of EVs was 3.5*10^4^ particles/mL

Experimental conditions were optimized to obtain excellent performance of the sensor, containing the concentration of Cas13a, T7 RNA polymerase, CD63 aptamer, and SA-MB, the reaction time and temperature of Cas13a. The reaction time of Cas13a was studied in the range from 10 to 60 min. As shown in Fig. 4A, the maximum fluorescence intensity was observed at 40 min, suggesting that 40 min was the optimal reaction time of Cas13a. In Fig. 4B, the fluorescence gradually increased with the increase of Cas13a temperature, but rapidly decreases in the range of 37–51℃. Therefore, 37 ℃ was chosen as the ideal temperature. As shown in Fig. 4C, fluorescence intensity gradually increased with the Cas13a concentration in the range from 0 nM to 100 nM, and then reached a maximum at 100 nM. Thus, 100 nM was adopted as a reaction concentration of Cas13a. The optimization of concentrations of T7 RNA polymerase is shown in Fig. 4D, where 50 U was selected as the optimal concentration of T7 RNA polymerase because the fluorescence signal reaches the maximum at 50 U of concentration of T7 RNA polymerase. As shown in Fig. 4E, 5 μM was selected as the optimal concentration of CD63 aptamer because maximum fluorescence occurred at 5 μM of CD63 aptamer concentration. For the optimization of SA-MB amount shown in Fig. 4F, fluorescence intensity gradually increased with the increase of SA-MB amount from 0.1 to 1 mg/mL, and reached the maximum at 1 mg/mL. Therefore, 1 mg/mL was adopted as the optimal amount of SA-MB.

**Fig. 5:**
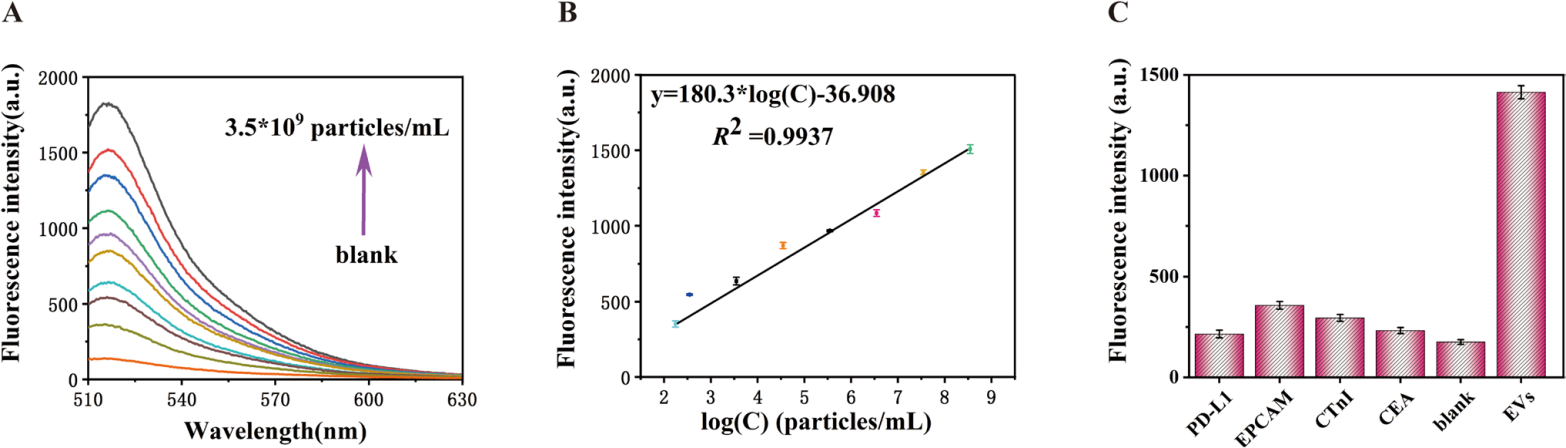
**A** Fluorescence spectra of EVs at different concentration (from bottom to top: blank, 175, 3.5*10^2^, 3.5*10^3^, 3.5*10^4^, 3.5*10^5^, 3.5*10^6^, 3.5*10^7^, 3.5*10^8^, 3.5*10^9^ (particles/mL). **B** Linear relationship between fluorescence intensity and logarithm of EVs concentration with a base of 10. a.u. arbitrary units. **C** Investigating the selectivity of this biosensor. Relative fluorescence responses initiated by EVs and other interferential proteins including PD-L1, EpCAM, CTnI, CEA, and blank control. The concentration of EVs was 3.5*10^8^ particles/mL. The error bar represents the standard deviations of measurements (*n* = 3)

To obtain high sensitivity, reaction concentrations of T7 Blocker were also investigated. As shown in Fig. S1, the fluorescence intensity reaches its maximum value when the concentration of T7 Blocker is 7.5 μM. Thus, 7.5 μM was used as the optimal amount of T7 Blocker. In addition, the reaction concentration of Apt 63 can also affect the fluorescence signal intensity. It can be seen in Fig. S2 the corresponding fluorescence intensity reaches the plateau when the concentration of Apt 63 is 0.5 μM. Therefore, we chose 0.5 μM as the optimal concentration of Apt 63 for the followed study.

### Sensitivity and specificity

Under the optimal experimental conditions, the analytical performance of the fluorescence biosensor was investigated using EVs of different concentrations. The fluorescence intensity exhibited an upward trend corresponding to the escalating concentration of EVs, spanning from 0 to 3.5*10^9^ particles/mL. A good linear relationship between fluorescence signal and logarithm of EVs concentration was observed in the range of 175 to 3.5*10^9^ particles/mL (Fig. 5B). The regression equation is expressed as y = 180.3*log(C) – 36.908 (*R*^2^ = 0.9937), where y represents the fluorescence intensity and C is the concentration of EVs. According on the 3δ rules, low limit of detection (LOD) was approximately 60 particles/mL (approximately 1 zmol/L). A comparison with other methods for EVs detection (Table S2), where the proposed fluorescence biosensor presents a relatively lower limit of detection and a broader linear range, suggests its outstanding performance. Briefly, superior sensitivity is attributed to the powerful T7 RNA polymerase and CRISPR/Cas13a enables multiple amplifications as well as the high sensitivity of the fluorescence platform.

Moreover, the selectivity of the sensor was assessed using PD-L1, EpCAM, cTnI, and CEA as interferential proteins (1 μg/mL). As shown in Fig. 5C, fluorescence intensities for PD-L1, EpCAM, cTnI, and CEA are similar to those of the blank control and can be ignored. However, significant fluorescence response was observed only in the presence of EVs (3.5*10^4^ particles/mL). These results indicate the potential of this method for accurate identification and monitoring EVs in complex samples. In addition, to validate the accuracy and precision of the method, three different concentrations of EVs samples were prepared by diluting with PBS. As shown in Table S3, the recovery rate varied from 93% to 106 %. The above results indicated that the sensor had good accuracy for the detection of EVs.

### Application in clinical samples

**Fig. 6:**
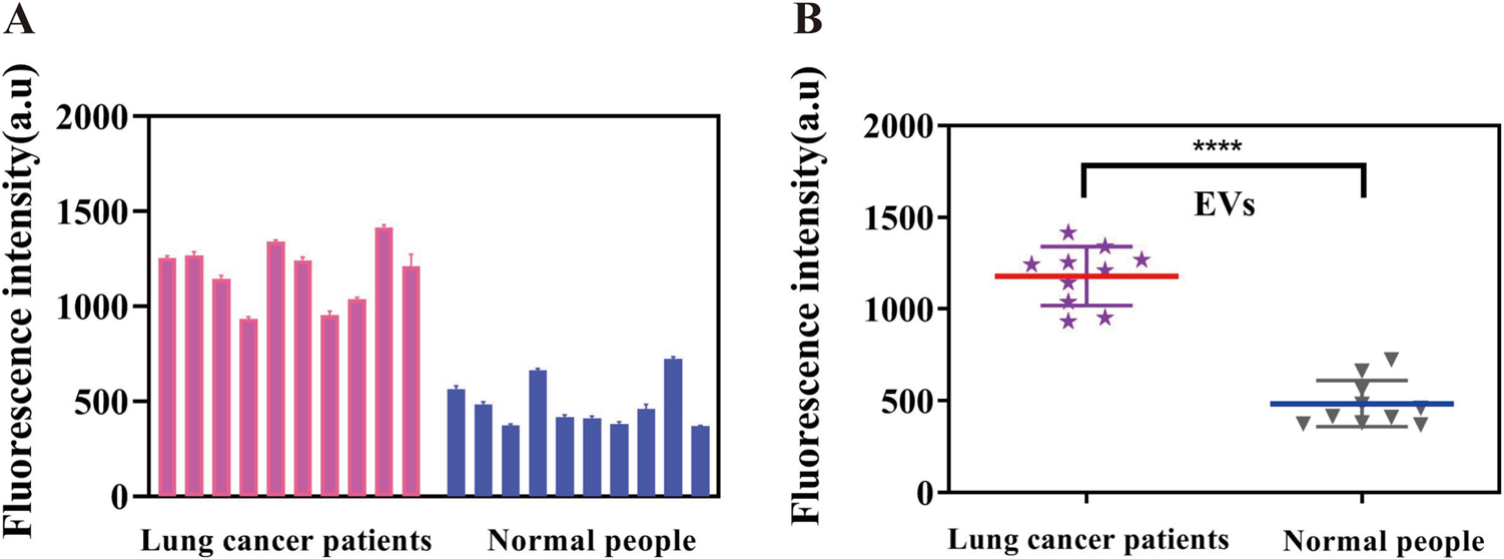
Clinical sample analysis. **A** Measurement of EVs in plasma from 20 clinical samples using the proposed sensor. **B** There is a significant difference of EVs expression levels between lung cancer patients (*n* = 10) and normal people (*n* = 10). (****p < 0.0001). The concentration of EVs was 3.5*10^8^ particles/mL. The error bar represents the standard deviations of measurements (*n* = 3)

To evaluate the application potential of the sensor in clinical samples, exosomes derived from human plasma were extracted for detection. As shown in Fig. 6A and B, the fluorescence intensity response to EVs derived from plasma of lung cancer patients was significantly higher than that of healthy individuals (*p* < 0.0001), indicating higher EVs in plasma from lung cancer patients. These results suggest that EVs may be potential biomarkers for lung cancer, and also that our method is a promising tool for EVs detection in clinical samples for diagnosis of lung cancer.

## Conclusions

In this work, high sensitivity and specificity of EVs detection were achieved. This method takes full advantage of assisting the specific recognition between the EVs biomarker and its aptamer, magnetic separation technology, the signal amplification strategy based on T7 RNA polymerase, and the crRNA-directed RNA cleavage ability of the CRISPR/Cas13a. Magnetic separation technology is used to eliminate the interference of complex matrix and improve the detection efficiency of EVs, and the introduction of T7 RNA polymerase and CRISPR/Cas13a achieves multiple amplification of sensor signals, thus improving the accuracy and sensitivity of the method. Consequently, LOD for EVs is low down to 60 particles/mL (approximately 1 zmol/L), with a linear range from 175 to 3.5*10^9^ particles/mL. Through the validation of clinical samples, our method can accurately distinguish patients with lung cancer from healthy people, so it can be used as a sensitive and effective tool for cancer diagnosis. Even more fascinating, our method may be extended to the detection of other biomarkers by selecting appropriate aptamers. In short, this study provides a simple and efficient fluorescence method for the detection of EVs, which has ideal sensitivity and accuracy, and can meet the application of cancer diagnosis.

## Supplementary Information

Below is the link to the electronic supplementary material.

Supplementary file1 (DOC 637 kb)

## Data Availability

Data can be provided according to journal requirements.
