# Supplementary material for: An ultrasensitive sensing strategy based on CRISPR/Cas13a and T7 RNA polymerase amplification for detection of extracellular vesicles
Source: Anal Sci. 2025 Jul 17;41(10):1627–36. doi: 10.1007/s44211-025-00828-3 (PMC12464105; doi:10.1007/s44211-025-00828-3)
Supplement: Supplementary file 1 — Supplementary file1 (DOC 637 kb) [file 44211_2025_828_MOESM1_ESM.doc]

***Supporting information***

**An ultrasensitive sensing strategy based on CRISPR/Cas13a and T7 RNA polymerase amplification for detection of extracellular vesicles**

Fengying Ran ^1,2^, Huimin Huang^1,2^, Bing shang ^3^, Weidong Peng^1^, Lun Wu^1^, Kang Ling^2^, Xiaoyu Xie^1^

^1^School of Pharmacy, Health Science Center, Xi’an Jiaotong University, Xi’an 710061, Shanxi, China

^2^Sinopharm Dongfeng General Hospital, Hubei University of Medicine, Shiyan 442008, Hubei, China

^3^Department of Pharmacy, National Cancer Center/National Clinical Research Center for Cancer/Cancer Hospital, Chinese Academy of Medical Sciences and Peking Union Medical College, Beijing, 100021, China.

**1.** **Characterization of** **EVs**

**1.1. Transmission electron microscope(TEM)**

15 μL **EVs** suspension was placed on 400 mesh carbon membrane copper mesh, fixed with 2% glutaraldehyde, dyed with 2% phosphotungstic acid for 10 min. Washed twice with PBS, dried at room temperature, and the morphology of exosomes in the copper mesh was observed at 80 kV voltage.

**1.2. Nanoparticle tracking analysis(NTA)**

Nanoparticle tracking analysis can be used to observed and quantified the **EVs** in the suspension. 20 μL of **EVs** were diluted 100 times with PBS. The diluted **EVs** were observed after filtering through 0.22 μm membrane. When the sample is irradiated by laser in NTA, the scattered light signal caused by Brownian motion of **EVs** nanoparticles can be collected by optical microscope and SCMOS camera with optimized parameters, and the concentration and particle size distribution of **EVs** can be measured at the same time.

**1.3. Western blots(WB)**

**EVs** were lysed in buffer containing protease inhibitors, and protein quantification was performed using BCA kit. The denatured proteins were separated by sodium dodecyl sulfate polyacrylamide gel electrophoresis (SDS-PAGE,12%), cooled to room temperature, transferred to polyvinylidene difluoride membrane (PVDF), and blocked with 5% (w/v) bovine serum albumin in TBST for 1 h. Further immunoblotting was performed with primary antibody CD63 and β-actin in blocking buffer at 4℃ overnight. Finally, the second antibody coupled with HRP was incubated at 37 ℃ for 1 h. The membrane was rinsed 3 times with TBST buffer and imaged by gel imaging system.

**2. Polyacrylamide gel electrophoresis(PAGE)**

12% polyacrylamide gel electrophoresis (PAGE, 30% acrylamide-bisacrylamide 4 mL,10×TBE 1 mL, H2O 4.95 mL, TEMED 5 μL, 10% APS) was prepared to verify the feasibility of T7 promoter induced transcription amplification. The 10 μL sample was mixed with 2 μL 6 × Glycerol loading buffer and added into the gel well. The buffer for electrophoresis was 1 × TBE. The constant voltage was 120 V, the electrophoresis time was 90 min, and then the gel was soaked with 1 × 4SGelRed dye solution for 30 min, and placed under the gel imager for imaging.

**Table S1. Sequences and involved oligonucleotide probes**

| Name | Sequence |
| --- | --- |
| CD63 aptamer | 5’**ATATACACCCCA**CCTCGCTCCCGTGACACTAATGCTATTTTTT-Biotin3’ |
| Aptamer Blocker (T7 promoter) | 5’ **TGGGGTGTATAT** TAATACGACTCACTATAGGG3’ |
| DNA templet | 5’CTACCTGCACTGTAAGCACTTTGCCCTATAGTGAGTCGTATTA**ATATACACCCCA3’** |
| RNA transcript | 5’GGGCAAAGUGCUUACAGUGCAGGUAG3’ |
| CrRNA | 5’GAUUUAGACUACCCCAAAAACGAAGGGGACUAAAAC-CUACCUGCACUGUAAGCACUUUG3’ |
| ssRNA-FQ probe | 5`6-FAM-UUUUUU-3`BHQ1 |

**Table S2. Comparison of this paper with other methods for EVs detection**

| Number | Analytical Method | Linear range  (particles/mL) | Detection limit  (particles/mL)/(Zmol/L) | Refs |
| --- | --- | --- | --- | --- |
| 1  2  3  4  5  6  7  8  9  10  11 | Electrochemical impedance spectroscopy(EIS)  Electrochemical impedance spectroscopy(EIS)  Electrochemical  Electrochemical  Colorimetry and photothermal  AuNPs amplified SAW immunosensor  Surface-enhanced Raman spectroscopy (SERS)  Surface plasmon resonance (SPR)  Fluorescence  Fluorescence  Fluorescence | 2.03× 10^3^-2.03× 10^9^  1× 10^9^-3× 10^9^  7.9× 10^6^-3.17× 10^8^  1× 10^7^-1× 10^10^  2× 10^6^-4×10^7^  1× 10^3^-1× 10^9^  1 × 10^4^-5 × 10^6^  5 × 10^3^-6.5 ×10^7^  1×10^5^-1×10^10^  500-5× 10^4^  175-3.5× 10^9^ | 2.03× 10^3^/34  1× 10^8^/1.7× 10^6^  3.43× 10^6^/6× 10^4^  7.83× 10^6^/1.3× 10^5^  1.03× 10^6^/1.7× 10^4^  1.1×10^3^/18  5.3 × 10^3^/88  5 × 10^3^/83  4.21 × 10^4^/702  100/1.7  60/1 | [1]  [2]  [3]  [4]  [5]  [6]  [7]  [8]  [9]  [10]  **This work** |

**References**

[1] J. Zhang, Q. Chen, X. Gao, Q. Lin, Z. Suo, D. Wu, X. Wu, Q. Chen, A label-free and antibody-free molecularly imprinted polymer-based impedimetric sensor for nsclc-cells-derived exosomes detection, Biosensors-Basel. 13 (2023)647.

[2] J. Tu, J. Min, Y. Song, C. Xu, J. Li, J. Moore, J. Hanson, E. Hu, T. Parimon, T.Y. Wang, E. Davoodi, T.F. Chou, P. Chen, J.J. Hsu, H.B. Rossiter, W. Gao, A wireless patch for the monitoring of c-reactive protein in sweat, Nat. Biomed. Eng. 7 (2023) 1293-1306.

[3] H. Zhang, X. Zheng, T. Zhao, Y. Chen, Y. Luo, Y. Dong, H. Tang, J. Jiang, Real-time monitoring of exosomes secretion from single cell using dual-nanopore biosensors, ACS Sens. 8 (2023) 2583-2590.

[4] J. Lu, M. Wang, Y. Han, Y. Deng, Y. Zeng, C. Li, J. Yang, G. Li, Functionalization of covalent organic frameworks with dna via covalent modification and the application to exosomes detection, Anal. Chem. 94 (2022) 5055-5061.

[5] X. Zhang, X. Zhu, Y. Li, X. Hai, S. Bi, A colorimetric and photothermal dual-mode biosensing platform based on nanozyme-functionalized flower-like dna structures for tumor-derived exosome detection, Talanta. 258 (2023) 124456.

[6] C. Wang, C. Wang, D. Jin, Y. Yu, F. Yang, Y. Zhang, Q. Yao, G. Zhang, Aunp-amplified surface acoustic wave sensor for the quantification of exosomes, ACS Sens. 5 (2020) 362-369.

[7] J. Wang, H. Xie, C. Ding, Designed co-dna-locker and ratiometric sers sensing for accurate detection of exosomes based on gold nanorod arrays, ACS Appl. Mater. Interfaces. 13 (2021) 32837-32844.

[8] Q. Wang, L. Zou, X. Yang, X. Liu, W. Nie, Y. Zheng, Q. Cheng, K. Wang, Direct quantification of cancerous exosomes via surface plasmon resonance with dual gold nanoparticle-assisted signal amplification, Biosens. Bioelectron. 135 (2019) 129-136.

[9] H. Cui, T. Zheng, N. Qian, X. Fu, A. Li, S. Xing, X.F. Wang, Aptamer-functionalized magnetic ti(3)c(2) based nanoplatform for simultaneous enrichment and detection of exosomes, Small. (2024) e2402434.

[10] P. Yi, D. Luo, Z. Gao, Q. Chen, Y. Zhou, Fluorescent aptasensor based on the mnps-crispr/cas12a-tdt for the determination of nasopharyngeal carcinoma-derived exosomes, Mikrochim. Acta 190 (2) (2023) 74-83.

**Table S3. Recovery of EVs spiked to PBS (a means values of three measurements)**

| Add **EVs**  (particles/mL) | **EVs** found  (particles/mL) | Recovery(100%) | RSD(%) |
| --- | --- | --- | --- |
| 350  350000 | 324^a^  338844^a^ | 93  97 | 3.26  2.26 |
| 35000000 | 37153523^a^ | 106 | 2.27 |

**The optimization of various reaction conditions.**

**
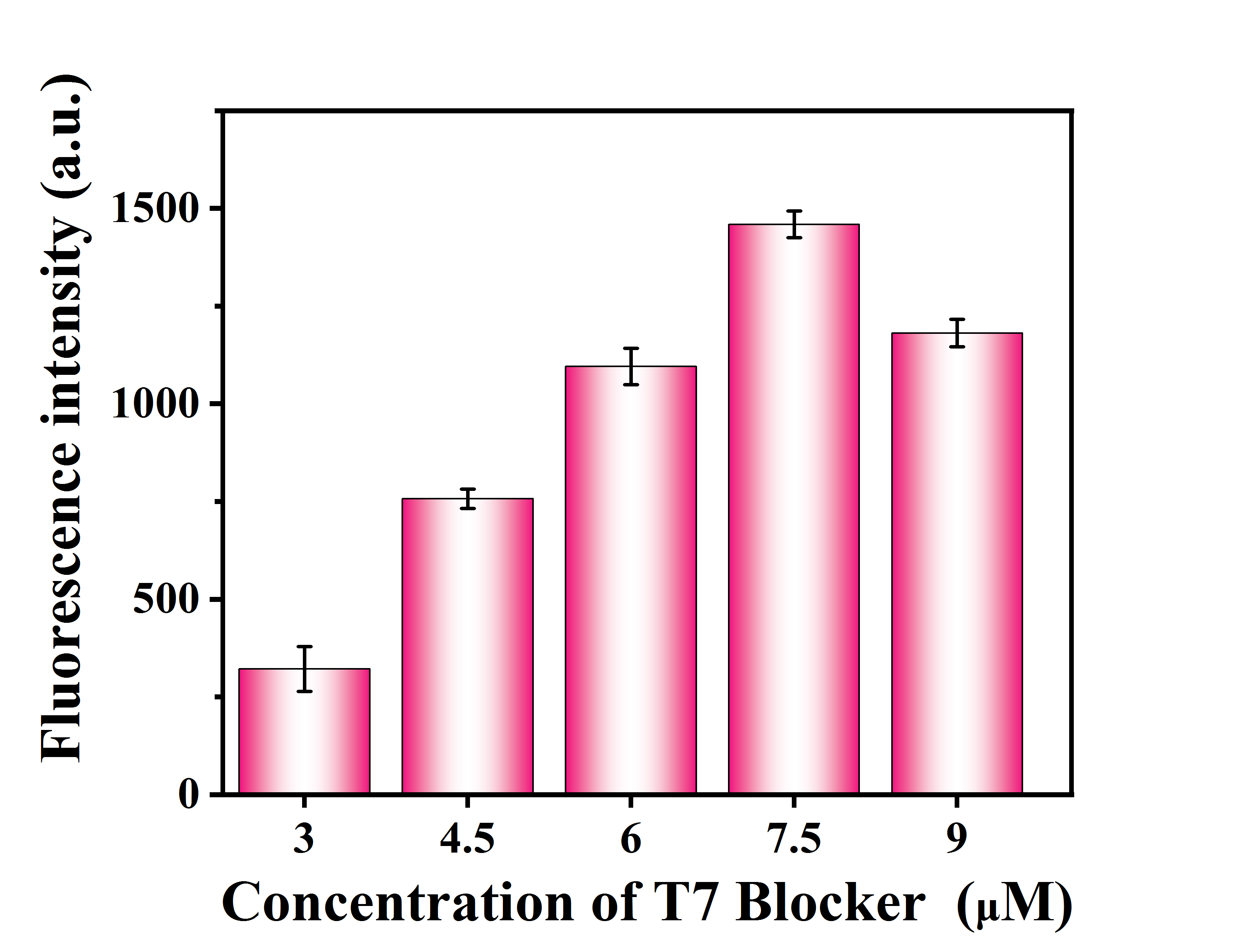
**

Fig. S1. With the various concentration of T7 Blocker.


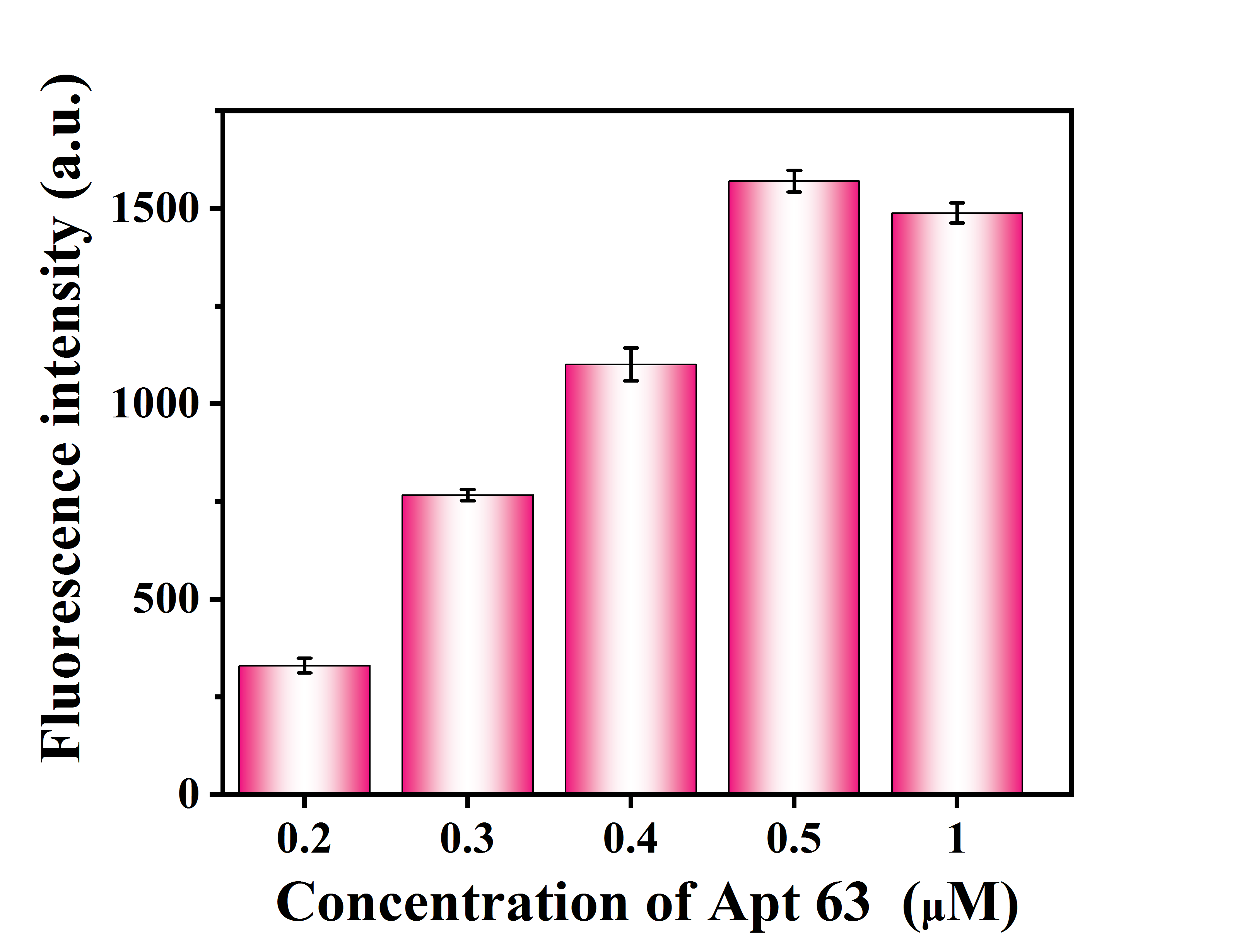


Fig. S2. different concentrations of Apt63 attached to SA-MBs. The concentration of EVs was 3.5*10^4^ particles/mL, SA-MBs: 1 mg/mL. The error bar represents the standard deviations of measurements (n = 3).
